# Illustrations of the Enquiry Respecting Tuberculous Diseases

**Published:** 1823-03-01

**Authors:** 


### VIII.

*Illustrations of the Enquiry respecting Tuberculous Diseases.* By John Baron, M.D. Physician to the General Infirmary at Gloucester. Octavo, pp. 236. five plates, coloured. London, Dec. 1822.

Throughout the preface to this volume we think we can perceive a tone of querulous animadversion on modern medical investigations—on the modes in which researches are carried on—on the validity of medical testimony and medical records—on the claims to improvement which the moderns have set up over their forefathers—and on the want of logical precision in our language and reasonings. That there is room for complaint, and still more for regret, on all these points, we are ready to acknowledge; but as the cause of these evils lies more in the difficulty of the subject than in the negligence or incapacity of the investigators, we cannot join in this declamation against the latter. We are really tired of the exhortations to medical logic and mathematical precision, when the *things* themselves are but imperfectly known, (many of them totally unknown,) and where language and terms must alter with every advance or discovery, whether of error or truth. Let these complainers look at the science of chemistry, which may be termed an exact science. How often have the theories, the terms, and the language altered and vacillated, even in the short space of twenty or thirty years! The science of medicine is a thousand times more difficult than that of chemistry, because we have not only the laws of physics but the laws of life to study—most of the latter entirely beyond our ken, and few of them as yet understood. Again, as it is only from those who are engaged in the busy scenes of actual practice that we can hope for improvements in medical science, how are we to expect all that rigid mathematical exactness and logical precision which are the products of intellectual labour in the cabinet, rather than the offspring of observation at the bed side. We consider it indeed as yet the age for accumulating facts rather than for arranging deductions; and therefore we firmly believe that hypercriticism on the modes of recording those facts and observations which serve as the basis of our science, are more calculated to retard than to accelerate its progress. We confidently appeal to all those who know the difficulties of medical investigations—the drudgery of practice—the shortness of time—the anxieties and distractions of life—and the thousand obstacles to abstract reasoning, whether one in a thousand can either prosecute or profit by studies attended with such exquisite refinement, mathematical correctness, sceptical scruples, and laws of logic, as a few modern reformers are labonring to introduce upon all occasions, whether of theory or practice.* This much we know, that it is labour

* Even the "great Haller," whom our author has eulogized, (and we wish not to detract from his merits,) was evidently convinced that *very few* indeed could proceed on these nice steps so warmly recommended by himself and our present author. We give the quotation which Dr. Baron himself has brought forward. "Est in his omnibus ars quædam inveniendi, quæ breviter dici non potest, *et quam paucis mortalibus*

in vain. They are incapable of effecting the refinements they propose; and if they were, they would have few disciples among the great mass of the profession. We confess it, (perhaps it ought to be with shame) that we are among those who are not "too fond of the right to pursue the expedient;" for we have rarely seen any practical good result from attempts to carry this "*beau ideal*" into execution. It must be by patient industry, close observation, and plain philosophizing, that medicine can be rescued from empiricism or conjecture.

"How different," says our author, "were the feelings and principles which actuated the minds of some of the distinguished men who have, in times past, successfully cultivated either physiological or pathological science, from those which are most generally adopted at the present day." P. xxi.

We much doubt whether our forefathers had a bit more zeal, or better means, or honester intentions than ourselves. True it is there were fewer writers among them, from their being compelled, in general, to write in a learned language; consequently, there was then a great deal less of the trash which now daily issues from the toiling press. But, on the other hand, there was far less attention paid throughout the great mass of medical society, in those times, to *pathology* which, as connected with close observation of symptoms at the bed-side, must form the firm basis of all our useful knowledge.

We shall now quit this subject, and endeavour to convey to our readers as clear an analysis of the work before us as we are able—and that without prejudice or partiality.

This illustration is divided into six chapters, three of which, viz. the first, second, and sixth, will principally occupy our attention—the other three being chiefly a series of criticisms on modern and ancient writers. The first chapter is "on the Progress of Pulmonary Tubercle;" and, as our author thinks that it will facilitate the understanding of what he has here to advance, if he briefly recapitulates some of the propositions set forth in his printed "Enquiry," we shall give the recapitulation in his own words.

"First, then, I affirm, 'That tubercles exist in almost every

---

*natura concessit.* Oportet absque prejudicio ad opus venire, non eo animo ut videas, quæ classicus auctor descripsit; sed ea cum voluntate, ut ea videas quæ natura fecit." Now we firmly believe that the modern physician comes to the interrogation of Nature with *fewer prejudices* than did the ancient; besides the advantages of an accumulated stock of knowledge transmitted by record.

texture of the body, and that their origin and essential character will probably be found to be the same, wherever they are discovered.'*

"II. That tubercles in their commencement, are small vesicular bodies, (i. e. hydatids,) with fluid contents.†

III. That these bodies subsequently undergo transformations, on the nature of which their tuberculous character depends; that these transformations are progressive, but not uniform, and that it is only in the larger bodies of this kind that they can be accurately traced. That they commence with an opake spot, which advances with different degrees of rapidity, and ultimately converts both the contained and containing parts into substances very different from what they were at first.‡

"IV. That on the size and relative position and structure of the tubercles, which are thus formed, depend the characters of many of the most formidable disorganizations, to which the human body is exposed.§

"V. That considering the transmutations, which these bodies undergo, the condition in which they may be found will be modified by the time at which they may happen to be examined.‖

"VI. That it is rarely that we can have an opportunity of seeing the first steps of these morbid phenomena in the human subject, because the tubercles are generally formed, and the elementary character of course lost, before death permits us to make enquiries respecting altered or morbid structure.¶

"VII. That some tumours are formed by the aggregation of tubercles, and that the characters of such bodies are materially influenced by the relative position and contents of the elementary parts, of which they may happen to have been composed, or in other words, that 'varieties in the arrangement of the elementary parts of morbid growths, will of course cause corresponding varieties in their appearance.'[a]

"VIII. That, therefore, diversity of appearance in tubercles or tumours does not imply diversity of origin, for it has been demonstrated that substances and textures of very different properties may be found even within the same cyst, thereby merely denoting different gradations in the changes, to which these bodies are liable.[b]

"IX. That the disorganizations above referred to are not the product of any species of inflammation, and that though inflammation may attend their growth, and modify the symptoms, which they occasion, yet that it is very different both in its origin and consequences from that species which attacks a part unaltered by previous disease; that in the first instance it is to be considered as the consequence, and in the latter as the cause of altered texture."[c]

It is necessary to bear in mind that the morbid appear-

---

* Vide Enquiry, p. 75. † Id. p. 214. ‡ Id. p. 93. *et cet. loc.*
§ Id. p. 215. ‖ Id. p. 217. ¶ Id. p. 241.
[a] Id. p. 218. *et cet. loc.* [b] Id. pp. 221, and 231. [c] Id. p. 120.

ances may be very different, according as a small or a great number of tubercles are evolved. In the *former* case they generally attain a larger size than in the *latter*—and then they may either produce a vomica, or a tumour, or both. In the latter case, (where a great number are evolved,) if they advance simultaneously, no one can much outstrip the other in growth, in which case we usually see a great number either approximating or in actual contact, and with qualities varying according to the nature and period of their progress—and it is this progress in the common tubercular phthisis, that we are now to attempt to trace.

In the earliest stage of pulmonary tubercles they are incognizable by the touch, on account of their delicacy and elasticity; but they are visible as small vesicular transparent bodies shining amid the unchanged surrounding texture.

"Should any of them happen to have been generated on the surface of the membranes, they there may be seen clustering together, and resemble both in size and general character the beautiful globular incrustations, which beset the stalks and leaves of the ice plant." P. 10.

In the human subject it is rare that tubercles can be seen in this their primitive state; but only at a somewhat later period of their progress. At this period the softness and delicacy of the vesicle is lost, its transparency diminished, and its size increased. On examining the lung where they exist, a distinct granular sensation is communicated to the fingers.

"The progress from this period is evinced by an augmented size, a firmer texture, and a complete loss of transparency, a yellow opake body being perceptible. In this state they sometimes fall into ulceration and prove fatal. But before such an event takes place, it occasionally happens that many of them advance further and exhibit other appearances. Except where they are in contact with each other, they go on increasing in bulk. The coats of some become thick and hard, and almost cartilaginous; while their contents may vary both in colour and consistence. Others proceed in a different way, and are condensed into solid bodies of an uniform texture, the cysts and the containing parts being scarcely discernible from each other." 11.

The appearances in those who die in this state, are as follow:—some tubercles will be found firm and solid—others with thick dense coats, containing curdy, cheesy, or purulent-looking substances: others, again, will appear partly destroyed by the progress of the ulceration, exhibiting only a firm, almost cartilaginous remnant of emptied cyst, conspicuous among the surrounding disease. "Should a great number of contiguous tubercles have fallen into this state, deep and extensive and irregular shaped fissures and excavations are thereby formed"

"In the progress of the tuberculous disease, there are corresponding changes in the surrounding lung, which it is necessary now to note. At the first development of tubercles, whether in the lungs or elsewhere, the surrounding texture seems to undergo little or no alteration. The lung retains its fresh pink colour, and its light elastic feel, and there appears to have been no interruption either to the circulation of the blood or air.

"As the tubercles increase in size and in density, and approximate each other, they cause greater disturbance in the system. The blood is impeded in its circulation, and respiration is of course rendered quick and laborious on slight exertions. The consequences are obvious, the lung becomes firmer and of a darker colour, and ultimately exhibits that appearance, which has been supposed to be indicative of a particular species of disease.*" 13.

This dark and indurated state of lung is occasionally obliterated by the increase and coalescence of tubercles into a dense and solid structure, with here and there a trace of the original tuberculous character, to the total exclusion of every thing like the pulmonic texture.

"The changes of structure above described, are indicated by corresponding symptoms. Tubercles, in their incipient state, may exist without producing much disturbance in the system, and they may pass onwards towards consolidation, if they be not very numerous, without affording almost any signs of their existence; and in this consolidated state they may continue, and not in any material degree tend to abridge life. The unexpected occurrence of solid tubercles or tumours in the lungs of those, who had not previously manifested any symptoms of such disease, bears me out in this assertion. When tubercles are fully consolidated, there is the strongest reason to believe that they do not subsequently fall into a state of suppuration. This occurs chiefly in those that were not destined to arrive at this point.

"The consolidation therefore just referred to, may in some measure be considered as a favorable termination to tubercles, as life has been found to be compatible with their existence, except in cases where they occupied a large proportion of the lung, or produced accretion of the membranes. It is in that period of their progress, which is intermediate between the state last mentioned, and their first developement, that all the symptoms, characteristic of tuberculous phthisis occur. This will be apparent by attending briefly to the ordinary progress of the disease." 15.

It is well known that, in a person who has tubercles, we

---

* Hepatization.

find cough at intervals, without expectoration, but with occasional oppression about the chest, and hurried breathing on slight exertions.

This state may exist, at intervals, for many months, or even years, without any other sign of disease. A catarrh may encrease these symptoms, or render them more permanent, and then some yellowish or whitish expectoration may appear, mixed with blood—or a gush of blood may precede the occurrence of expectoration. Our author has known this hæmorrhage repeatedly happen and ultimately prove fatal, where there was great consolidation of the pulmonary tissue by tubercles, and yet, where there was never any expectoration of the tubercular matter itself. It is from this, and other kindred cases, that he infers, that tubercles, once consolidated, do not subsequently suppurate or ulcerate.

Such expectoration as above described is, he thinks, a sure token of a tuberculous disease. One of them has given forth its contents, and more may do the same thing, though at intervals more or less extended, with proportionate recoveries for the time being. There may even be ultimate recovery after successive events of this kind, shewing, Dr. B. thinks, that there either were not a great number of tubercles in a state to undergo the ulcerative process, or that the tubercles were brought by accident or treatment into a quiescent state, and subsequently consolidated. It is comparatively seldom, however, that this termination is obtained either by nature or art.

Our author observes, that the matter expectorated is of different kinds—that from excited mucous surfaces being different from the contents of a tubercle—and this last differing materially from the purulent secretion that issues from the ulcerated internal surface of a tubercle after it has discharged its contents. The appearance of pus, therefore, by no means necessarily indicates the presence of tubercles, "as is generally supposed."*

The dark-coloured induration of the lung before alluded to, does not attend tubercles in their early state, though it more or less accompanies them as they advance. It readily falls into decay when the enveloped tubercles are undergoing a process of dissolution. When this induration occurs to any extent, it occasions or increases the difficulty of breathing,

* We are not aware that medical men generally suppose that where pus is, there a tubercle must be. Nothing can be more common than the knowledge that pus may come even from an unbroken surface, when it is strongly excited.

and there is generally a livid appearance about the lips and countenance.

Great variety occurs in respect to pain. Sometimes there is little or none throughout the disease—"at other times the disruption of every successive tubercle seems to be accompanied by deep-seated and acute darting pains."

"Tubercles in the pleura cause sometimes effusion into the cavity, more frequently accretion. When the latter event takes place, there is cough and dyspnœa and a rapid pulse, but no expectoration. But when tubercles in the lungs, in a state of ulceration are added to it, we have in conjunction with the symptoms already enumerated, the expectoration of tuberculous matter, hectic fever, &c. The mode of breathing in cases, where accretion of the pleuræ has taken place, is different from what it is when the lungs are free within the cavity. In the first mentioned instance, 'the shoulders are drawn forwards, the ribs do not move as in the natural state, the whole chest heaves at once; and most of the muscles on the trunk of the body seem to be called into action.'* On striking the chest of a person in this state, the sound emitted is like that produced by the percussion of a solid body, very different from that which a healthy chest affords, or when disease exists in the lungs without accretion of the membranes." P. 21.

When a patient happens to be cut off by another disease, before the tuberculous affection has run its usual course, the same lung will sometimes present examples of all the progressive changes which our author has described in this, and his preceding Inquiry. An interesting case, in illustration, is given, and a plate from the same subject.

It is impossible to describe all the varieties of appearances which may arise from the peculiarities in the structure and arrangement of the tubercular masses; but, there is one which is so very common, that it requires to be noticed. It occurs when tubercles, originally soft and circular, grow in size and mutually press upon each others' boundaries. The globular character is thereby destroyed, and the divisions between each, so far as they can be traced, are angular, so that instead of circles, we have squares or figures of different kinds.

The extent to which tuberculous diseases may proceed without producing symptoms of pulmonary consumption, is much greater than could, *a priori*, be expected. Our author relates the case of a lady, who never exhibited any signs of pulmonary disease, the symptoms being rather those of an affection of the stomach and stricture of the œsophagus. Some weeks before she died, she was seized with symptoms of pneumonia.

---

* "Enquiry, p. 170."

Very frequent and large bleedings produced little effect. She expectorated pretty freely, but the dyspnœa became distressing, and she died. On examining the lungs, he found strong, but unlooked-for proofs of long existing disease. Almost the whole posterior portion of each lung was transmuted into a dense and nearly cartilaginous substance. Where the density was greatest, there the character of tubercles was obliterated; but it gradually re-appeared as they receded from this point, till, at last, their figure and boundary became perfectly distinct. All these appearances are accurately pourtrayed in the fifth plate. Two dissections are here given, shewing the progress of tubercles, from the smallest forms to the largest, in the pleura, liver, mesentery, and peritoneum.

Chap. II. This is on Tuberculous Diseases in the Inferior Animals.

Hitherto, in the present work, our author has kept out of sight his opinion respecting the *hydatidical origin* of tubercles, because, he was desirous that any degree of doubt which might still be attached to it, should not be permitted to obscure what may be demonstrated to occur in the *progress of tubercles*. This opinion, however, he has occasion incidentally to allude to, in tracing tuberculous diseases in inferior animals. Our author has selected the principal part of his descriptions from the work of M. Dupuy, an eminent French veterinary surgeon; his testimony being the more valuable, he observes, because it is, in a manner, extorted from him by irresistible evidence, in direct opposition to the main tenor of his work.

The *glanders*, as it is vulgarly called, is strictly a tuberculous disease attacking the lungs of the horse, and bearing the closest analogy to pulmonary consumption in the human subject.—*Farcy*, Dr. B. observes, is likewise a disease of the same genus, affecting another part of the animal. In glanders, the morbid appearances are not confined to the lungs. The nasal cavities, and the lymphatic glands, in many parts of the body, are also generally diseased.

We shall extract the following case from Dupuy, as our author thinks it bears strongly on his doctrine of the hydatid origin of tubercles.

" ' A cow, six years of age, was killed on the 2d of February, 1819. The body was examined immediately, and the following appearances were found. The pulmonary tissue was very much altered. It contained many cysts, enclosing hydatids of different magnitudes, from the size of a pea up to that of a goose's egg. Other cysts, of which the coats were of the consistence of cartilage, and even osseous, were filled with a substance analogous to that of bone; those which enclosed the hydatids were smooth and had the appearance of a mucous membrane. We thus found in these lungs hydatids and tuberculous matter, which would seem to prove, that these bodies, though very different in their physical qualities and their organization, have many affinities in common, in regard to the causes which determine their formation, and the manner in which these bodies alter the pulmonary tissue.' "* 47.

We shall not enter upon the train of commentaries which Dr. Baron has made on the above, and some other analogous cases. We cannot but think that comparatively few readers will feel interested in them.

Among domestic animals, none are more frequently affected with tuberculous diseases than sheep; and it was from these animals that Dr. Baron drew many of his descriptions in his former work. M. Dupuy has given many cases of this kind, from which our author has selected a few, and they will be found in page 60 of the Illustration.

Farcy has been thought, and apparently with justice, to bear the same relation to glanders, (which is the tuberculous phthisis of the horse,) that scrophulous affections of the extremities bear to the same disease in the human subject. Dr. B. thinks, that farcy is unquestionably connected with that condition of the system which gives rise to the formation of tubercles, whether in the lungs or elsewhere.

" Farcy seems clearly to be generated by disease of the lymphatics of the limb. The buds, as they are called, appear at first in the shape of small tubercles or buttons, which have the same organization, and go through the same changes that mark the progress of tubercles in the lungs. This disease seldom confines itself to the extremities. It generally advances towards the chest, attacks the membranes of the nose and lips, the glands under the jaw and in the neck, and finally developes itself in the shape of tubercles in the lungs, and then it constitutes the glanders. This, however, is not to be considered as the usual progress of the last-named disease, for it often begins in the lungs, and runs its course without any appearance of farcy whatever." 61.

In the third chapter, our author goes back to the father of physic, and, in the writings of the divine old man, thinks he sees almost all the knowledge of the moderns.

" The whole of what he has said respecting the diseases of the chest, bears wonderful testimony to the extent and accuracy of his observations, and it probably may demand all the knowledge that modern enquiries have unfolded, to enable us to do justice to his labours." 64.

---

* " De l'Affection Tuberculeuse, *par M. Dupuy*, p 269."

Our author has discovered, what was never denied, that Hippocrates used percussion and auscultation in exploring diseases of the chest. He seems in admiration at the extent of the Coan sage's knowledge when he points out the existence of tubercles in the ox, the dog, and the swine, what every butcher's boy in the island of Cos knew as well as Hippocrates. The divine old man's theory of the formation of tubercles from bile or pituita, our author considers as not a fair subject of criticism, therefore we shall say nothing on that head.

"It is quite sufficient to prove that he was aware of the existence of such bodies, *and of the consequences they produce.*" 64.

Now if Hippocrates was so well acquainted with the existence of tubercles, and the consequences they produce, he ought certainly to be acquainted with the *symptoms* which they give rise to. We ask Dr. Baron what kind of tubercle, and what stage of it, gives occasion for the following symptomatology of Hippocrates?

"Tuberculum in pulmone. Quum tuberculum in pulmone natum fuerit, tussis tenet et erecti cervicis spiratio, et dolor ad pectus acutus, et ad latera *et usque ad quatuordecim dies ita afficitur.* Nam plerisque per tot dies *tuberculi affectio inflammatur.* Sed caput dolet, et *videre non potest*, et corpus subvulvum fit, ac venis impletur." *De Morbis* II. *L.* 1.

We believe it would puzzle Dr. Baron or any other admirer of Hippocrates to find out what this fourteen day tubercle is which takes away the sight, and works so many other strange miracles. Our edition of Hippocrates not being the same as Dr. Baron's, we are unable to follow him through his numerous quotations, without a trouble which would be very badly repaid. Dr. Baron, if he wished his arguments to have as much effect as possible, might have avoided the pedantic display of so much Greek, and given the Latin version of Hippocrates, which would have been more generally understood. It is not, however, of very material consequence; for we apprehend the work will have a very limited circulation, except among the higher class of pathologists.

Dr. Baron considers it a remarkable thing that so few of the older writers should have mentioned these observations of Hippocrates. They are obscurely referred to by Galen, and entirely passed over by Celsus. In this we think they shewed their sense. There is no reason to believe that Sydenham had any accurate knowledge of tubercular consumption. In fact, we much doubt whether Sydenham ever opened a body in his life, with the view of ascertaining the seat of a disease—at least we have never seen any indication of post mortem researches in his works. It is rather more remarkable, however, that Boerhaave has no allusion to them in his aphorisms. Even Fothergill (Med. Obs. and Enq.) makes no mention of tubercular disorganization in his pathology of consumption. The following passage from the 38th epistle of Morgagni, makes it evident that the Italian pathologist had some opinions respecting the hydatid origin of tubercles corresponding with those of Dr. Baron.

" 'Finally,' he observes, 'read over again what I have formerly written to you, of hard granules or tubercles being prominent on the internal surface of the peritonæum or pleura; as water was even then extravasated in the great cavities, which those membranes surround, you will certainly find the series of successive changes, which I have described. It happened some years ago in a woman, that had been taken off by ascites; the external coat of the intestines was found to be distinguished with very frequent tubercles. Part of the small intestines was brought to me, that I might judge what these tubercles were. When I first examined them, they resembled small turgid lenticular glands; but they were without an orifice and solid, and seemed to be made up neither of a glandular nor of a fleshy substance, but to be of a middle nature, as it were betwixt both. I judged that I could determine on nothing more probable with regard to them, than to suppose that they were the remains of ruptured hydatids contracted into themselves, but not to so great a degree at present as to be dry and hard.' " 73.

As we fear we shall not be able to give such an account of the work before us as may be satisfactory either to the author or reader, (since it is almost entirely a tissue of medical polemics,) we shall here insert an extract, shewing Dr. Baron's matured creed and doctrine on the subject under review.

" On the whole, from the most attentive consideration of all that has been adduced now, as well as in my Enquiry, respecting peritonæal tubercles, or those which may be found in any other part, different though they may be in size and appearance, there can, I think, be no doubt as to the identity of their origin. All the general remarks which were formerly delivered, when we were treating of pulmonary tubercle, are applicable to kindred disorganizations, when they occur in the abdomen. A small number spread over the peritonæum, or in the mesentery, may exist without doing much injury. A greater number may produce universal accretion of the viscera, the extinction of the nutritive process, and a complete impediment to the functions of the alimentary canal. Should one or more of them grow from the ovaries or the uterus, or any other portion of the abdominal surface, they may increase till they attain a very great size, and then we may either have what has been denominated encysted dropsies, or ovarian dropsies, or tumours with different contents, and of different shapes, according to the transmutations which the hydatids may have undergone, or their collocation in the diseased structure. Again, should these bodies take their growth in any of the viscera, a series of the same changes may be detected, but the symptoms and the appearances will, of course, be modified by the part where they may happen to be generated." 79.

Into the 4th and 5th chapters, occupying nearly 120 pages, we shall not enter. They are entirely composed of criticisms on Bayle, Lænnec, Broussais, and Abercrombie. Dr. Baron weighs the opinions, statements, and even the words of those who come before his tribunal, with a balance so nice that we verily believe it would turn with the twentieth part of a minim of hydrogen gas! Heaven help medical authors, if Dr. Baron should ever conduct a medical review! He would grind them into dust. And, after all, for the life of us we cannot discover of what material consequence it is, whether a tubercle be originally a hydatid or a bit of cheese. We know that sometimes it grows larger, sometimes it remains stationary; sometimes it continues hard, sometimes it gets soft; sometimes its contents remain encysted, sometimes they break into the bronchia and produce purulent expectoration; sometimes these cavities heal, but much more frequently they continue to discharge, like an ill-conditioned ulcer, till death closes the scene. These things we know, and these things it is useful to know; but to enter into whole volumes of disputes respecting the origin of tubercles, (which, like the origin of all things, is veiled in mystery,) seems to us a waste of time that might be far better employed. The only thing of a useful or practical nature which we can see in the discussion is the question whether or not the growth of these tubercular disorganizations depends on a process of inflammation. Even this question does not appear to us to receive any light from the litigated point respecting the origin of the tubercle. Whether it be an invisible hydatid at the first moment of its formation; or an inorganized speck of some unknown material, still the process of its growth is to be decided (at least we think so) by far other evidence than that which is concerned in ascertaining its primitive form or nature. We shall give our author's sentiments respecting the question of inflammation.

"From a due consideration of all the phenomena of this, and other kindred diseases, I contend, that an inflammatory process is not that by which they are generated. There is the most conclusive evidence that cysts and sacs are not fashioned by the effusion of coagulable lymph around a diseased mass, or that the various appearances of these cysts, whether they be thick and firm, or slender and transparent—whether they be ossified, or show no signs of such a state—whether they contain a clear and watery substance, or a thick gelatinous purulent-looking matter, or be half-fluid or wholly so, or exhibit any of the above enumerated appearances in any variety of combinations—there is, I repeat it, the most conclusive evidence that such occurrences are not the result of any process analagous to inflammation. Further, I maintain, that whether there be one such body, or more than one, whether it be large or small, whether it be attached to the membranes or be imbedded in the brain itself, that all have one origin, and that it is common to the whole class of similar disorganizations in every part of the body." 188.

Our author guards his readers against confounding the growth of tumours themselves, with the effects produced by their presence in the surrounding structures. The latter may be of an inflammatory nature, while the growth of the tumour is of quite a different character. Indeed we have never been able to bring ourselves to the doctrine that the growth of a tumour, not even the pulmonary tubercle, was a species of inflammation. It appears far more probable that tubercles and tumours, especially of the encysted kind, are organized substances endued, unfortunately, with the power of drawing the nutriment of their growth from the surrounding parts, or from the blood itself. And we think it by no means improbable that their original or minutest cognizable form is that of a vesicle or hydatid, which may undergo many transformations in the progress of its growth, according to the structure in which it is placed, and various other circumstances. Nor is this doctrine inconsistent with the fact that, in tuberculated lungs, every catarrhal inflammation generally accelerates the growth of the tubercles; since it is evident that these last must draw the pabulum of their nourishment and growth from the neighbouring vessels, which vessels will be better able to afford this supply when gorged with blood, than when in their ordinary state of inactivity. The afflux of blood also to a tuberculated lung may act as a stimulus or excitement to that power by which the tubercle carries on its growth. This, we believe, is pretty nearly the doctrine of our author; and we really think that he would have had a thousand times more converts than he has, if he had taken far less pains to prove his point by such endless discussions and obscure reasoning—yes, obscure reasoning; for, notwithstanding all our author's attention to language, and censures on the language of others, we have seldom had occasion to peruse a work which has cost us so much labour to understand as the one now open before us. We can assure Dr. Baron that, however clear his ideas may appear to himself, he has not the happy art of always rendering them very clear to others. The import of the following passage, however, is sufficiently obvious.

"On the whole, I must repeat it as my conviction, that one grand error pervades our reasoning respecting structural diseases. We identify the changes which arise from the growth of bodies, originally foreign to the healthy structure of the animal, with those which arise from diseased actions of parts, where no previous change of structure had existed, and ascribe the change of structure itself, and the consequences which it induces, to one and the same cause. It was one great object of my Enquiry, to mark the boundaries between these two classes of diseases. It little concerns me, at present, to speak of the origin of the various adventitious bodies which alter the structure of animals; it matters not to what cause they are assigned, provided it be kept clear from those other disorganizations, which are the result of diseases, independent of any previous alteration in the healthy texture." 197.

Our author observes, that ever since the controversy between Ruysch and Malpighi, respecting the minute ramifications of blood-vessels, "inflammatory action" of some sort or kind has been held to be the sole agent in all deviations from the healthy structure of animals. In these respects, he justly remarks, we have doubtless overstepped the boundaries originally assigned to vascular action. The term "encysted abscess," as used by Dr. Abercrombie and others, our author affirms, and with no small shew of reason, to be "a contradiction in terms." Dr. Abercrombie, in describing one variety of suppuration in the brain, has called it—"a distinct abscess, confined within a soft cyst, the surrounding cerebral substance being healthy." To this language our author objects, and quotes the authority of Sauvages on the subject. He has no hesitation in affirming that these cysts are to the brain what vomica is to the lungs.

"The cyst, as I have more than once affirmed, is the part that is developed in the earliest period of the disease. It advances slowly in its progress, and may either retain its original character, or be gradually transmuted into a body with very different properties, which produces symptoms and consequences varying according to the structure and function of the part where it is generated." 201.*

*Chap.* VI. We must now occupy the few remaining pages of the article in the treatment of tubercular consumption, and that class of diseases.

Our author remarks that, in the inferior animals, the cir-

* In the process of softening down of a tubercle, without any thing like inflammation as a mean, our author seems to be supported by the observations of Lænnec, who has shewn that the liquefaction commences in the centre of the tubercle, where we cannot suppose any vessels go to carry on a process like inflammation.

cumstances which seem chiefly to predispose to the generation of tuberculous diseases, are, cold, moisture, and bad food. In the human subject we cannot doubt that the same agents produce similar effects. It is also but too well authenticated by wide experience, that when the animal frame has been deteriorated, from whatever cause, there is a disposition to transmit to the offspring of such being, whether man or inferior animal, the impaired constitution of the parent. In no class of diseases is the effect of hereditary taint more frequently observed than in pulmonary consumption. From the experiments of Dr. Jenner, detailed in our author's previous work, it appears that we can, by unsuitable food, soon call up a tuberculous disease in rabbits; and it is well known that a wet season and bad pasture will bring into existence the same disease, to a much greater extent, in sheep and other animals. It has also been ascertained that the disease in both cases may be got rid of (provided it has not too far advanced) by a more wholesome diet, and judicious removal from the influence of the other predisposing causes.

Dr. Baron is of opinion that the extent to which it is possible to excite the system to the absorption of morbid growths is much greater than is generally supposed. It is evident to every reflecting mind that our *methodus medendi* must entirely hinge on arresting the progress, or, if possible, causing the absorption of the tuberculous or foreign body, while, at the same time, we counteract the irritating effects of these tuberculous substances on the neighbouring parts. It is only at an early period of the disease we can hope to effect these purposes. Cough, therefore, and any of the other symptoms of pulmonic affection in a scrophulous or phthisical individual, should always cause alarm, and lead us to adopt precautionary measures:—even then it will often be too late, as disorganization, in its primary state, frequently exists without producing much disturbance in the system.

"Since it appears that whatever enfeebles the frame, or deteriorates the constitution, pre-disposes to the diseases in question, how shall we avert this pre-disposition? The answer is apparent: we must do every thing in our power to invigorate and fortify the tender frame; to bring all its functions into a healthy state, and by all means to endeavour to keep them so. But, suppose that this cannot be effected; that the pre-disposition has already advanced to incipient disease; that change of structure has actually commenced; what, in that case, is to be done? We must first seek the absorption of that change of structure; or, at all events, prevent its increase." 216.

What, it may be asked, are the agents by which we may effect this desirable object? We fear they are not very numerous. Mercury and alkaline preparations were formerly believed to be the most powerful deobstruents—and the former is unquestionably useful in many forms of diseases of the lymphatic system ; but in pulmonary tubercles its utility is more questionable. It appears to us that one great misfortune in this disease is, that we have, as it were, two contrary indications to pursue. We have to keep up the general system, and yet to obviate the effects of the tubercular irritation, which, in fact, is local inflammation in the parenchymatous structure of the lungs. It is obvious that the same means will seldom fulfil both these intentions. If then we could effect the absorption of the tubercles, we should do all that was necessary to cure the disease, anterior to ulceration. In a former page allusion was made to the connexion between external diseases of the horse, and some of the internal disorganizations, and it was affirmed that farcy bears the same relation to glanders, that scrophulous affections of the extremities or surface bear to pulmonary consumption in men. A practical inference is to be drawn from this connexion. If we possess any means of amending what is termed a scrophulous constitution, or of removing scrophula after it has once shewn itself, we have, Dr. B. thinks, sufficient grounds for believing "that what is efficient in regard to an external disorganization may be beneficial in counteracting an internal one."

"As far as my experience goes, there is no remedy which possesses such powers in promoting the absorption of morbid growths, as the hydriodate of potass. The reports of the influence of this remedy in curing bronchocele, as published by Dr. Coindet, of Geneva, first brought it to my notice. The nature of my own enquiries had led me anxiously to look for some agent of this kind ; and having been fully convinced of the affinity between bronchocele and the diseases of which I had treated in my Enquiry, I considered it extremely probable, that a remedy which could remove the first-mentioned species of disorganization, might be very beneficial in the others. On this principle I acted ; and the result of my trials has fully justified the anticipations which I had formed." 221.

Some cases are here detailed which we shall notice as the remedy has not, we believe, been tried in this country for the cure of pulmonary tubercles.

The first case was one apparently of physconia hydatidosa. The abdomen was as large as that of a woman in the last stage of pregnancy. The tumour, however, had more than once been reduced in size by the long continued use of mercury and Brandish's liquor potassæ, but it was never effectually removed.

"More than once its bulk was very much diminished, by an event which establishes its original character, and justifies the name which I have assigned to it: I mean, the disruption of one or more of its cysts, and the discharge of the contents into the alimentary canal; such fluids as hydatids are known to contain* in various stages of their progress, having, after the events just described, been discharged both from the stomach and per anum.

"This patient began the use of the hydriodate of potass in solution, on the 6th of October, 1821. She took at first eight drops twice a day, and continued them very regularly till the 23d of March, 1822. By this time a marked effect had been produced on the size of the tumour; but in consequence of some unpleasant feelings about the stomach and head, the drops were discontinued, and not resumed till June the 22d.

"From the use of this medicine, a very striking absorption of the diseased structure has taken place. Before she began it, the bulk was nearly as great as at any former period; now, it is not discernible by the eye; and it requires a pretty accurate examination by the touch to discover the remains of the substance, as she calls it, in the left iliac region." 222.

Dr. Baron thinks himself fully entitled to ascribe these results to the remedy in question; for excepting laxatives and the occasional application of leeches, no other remedies were employed.

The next case was somewhat a-kin to the foregoing, but in a person more advanced in life. The disease had been of slow growth; but not much regarded till a few months before our author was called in, when it gave great pain, and rendered the individual incapable of exertion. When Dr. B. examined her, he found a tumour, the size of a child's head, occupying chiefly the left side of the abdomen. It had a solid feel, and was very tender to the touch. Leeches, fomentations, and hemlock were directed; and then an ointment containing hydriodate of potass, to be rubbed upon the swelling night and morning. At the same time Brandish's liq. potassæ was administered internally.

"The rapidity with which the size of the tumour has been diminished, has quite surprised me. The remedies have not yet been used four weeks; and I am informed by the gentleman in regular attendance, that it does not equal half its original bulk. The pain and tenderness are quite removed, and the patient can walk, and exert herself almost as well as ever." 224.

Excepting in cases of bronchocele, our author has not seen any instances of absorption so rapid as this.

"In another case of a very formidable aspect, the efficiency of

---

* Vide Enquiry, p. 94.

the iodine, was shewn in a surprising degree. A gentleman had a series of tumours, which reached from the angle of the jaw to the top of the shoulder; some of them were very large, being equal in size to a goose's egg; they extended also to the front of the neck. Various powerful medicines, such as the compound calomel pill, liquor potassæ, &c. &c. were used without any effect. The hydriodate of potass was administered internally twice a-day, in doses of ten drops. It was continued for several months; at the end of which time, the morbid growths were almost completely removed, all that remained being a very small substance, not larger than the half of a walnut." 225.

The result of these cases encouraged our author to use the remedy in other diseases of a kindred nature; but the disorganizations were of such a description, as to preclude all rational hope, and therefore the results were disastrous. That there is strong reason to expect some effect from iodine, however, in the dissolution of pulmonary tubercles, Dr. Baron brings forward the following case, which we deem it necessary to give in the author's own words.

"A young gentleman, of a delicate frame, had been long affected with frequent cough; but at first he did not expectorate at all. He lost flesh; his pulse increased in velocity; his respiration was frequently hurried; and his countenance and manner indicated most serious disease. He had been in this situation for many months; when, after a fit of coughing, more violent than usual, a small globular-shaped, but somewhat flocculent mass of tuberculous matter, partially tinged with blood, was discharged. This event fully confirmed my suspicions respecting the cause of the harsh dry cough, which had so long harrassed him; and convinced me that tubercles existed in his lungs. Under circumstances of this kind, it is needless to say, that the most unfavourable prognostic was called for. I expected, of course, that in this, as in other similar cases, the patient would soon exhibit all the worst symptoms of pulmonary consumption. The expectoration of such matter as above described, having occurred a great many successive times, at considerable intervals, tended to strengthen my apprehensions. I dwell upon these particulars, because, without such proofs as they disclose, no fair estimate could be formed of the value of the remedy on which I chiefly relied for the removal of the complaint. I consider it, therefore, as proved, that the patient in question had tubercles in the lungs; and that they were rapidly hastening to that stage when recovery becomes almost hopeless.

"My plan of treatment was the following: I kept him in a regulated temperature; I stimulated the chest occasionally by blisters and tartar emetic, and confined him to a strictly vegetable diet. At the same time, anodynes were occasionally used, to abate the frequency of the cough. But knowing that all these means, unless the tubercles themselves could be got rid of, would be of little avail, I administered such remedies as appeared most likely to promote that object. I began with the use of Brandish's caustic alkali, in a little compound infusion of orange-peel, twice a-day. After employing these remedies for some weeks, I resolved to give him the hydriodate of potass. He began with eight drops twice a-day; and continued it for three weeks without intermission. It was then left off for about a fortnight, and resumed; the quantity having been increased to ten and twelve drops. The consequence of this treatment has been an almost complete removal of the cough; an entire cessation of all expectoration; a complete freedom of breathing; a reduction of the pulse to its natural standard; a healthy state of the stomach and bowels, and a decided augmentation of flesh and strength. The patient is able to take long-continued and active exercise on horseback, and has consequently been exposed to considerable alternations of temperature, without suffering inconvenience." 228.

Dr. Baron properly acknowledges, that the above case cannot yet be spoken of as definitively disposed of; but, so far as it goes, "it is perfectly satisfactory," and affords, he thinks, as strong a testimony as one case can give, that a most beneficial impression has been made upon the disease. "Should things continue to go on favourably, I shall feel no hesitation in believing, that this was an example of tuberculous phthisis, arrested in its progress mainly, I believe, by the medicine, of the qualities of which we have been speaking." He has exhibited it in several cases where ulceration had already taken place, and "all the most threatening symptoms of approaching dissolution existed." His trials in these cases confirmed what was to be expected—"namely, that the period for affording effectual relief was past." On the other hand, they have strengthened our author's conviction, that there is a period in the most formidable of all tuberculous diseases, when its course may be stayed, and the cause of subsequent evil eradicated from the system.

Our author takes this opportunity of recording some particulars of another case, which is applicable to the subject under consideration. The patient, a female, came under his care about three months previously to the date of report, affected with almost all the symptoms which characterize tuberculated accretions of the peritoneum, in a considerably advanced stage.

"There was weight, tension, hardness, and in some places tenderness of the abdomen; great oppression after taking food; almost constant nausea, and great irregularity in the functions of the bowels. There was likewise great emaciation and languor; a rapid, feeble pulse, and that peculiar anxious expression of the countenance, which I have elsewhere insisted upon as a strong indication of the internal disorganization mentioned above. In short, every symptom led me to believe, that that disease had actually begun to establish itself. My hopes of any essential relief, of course, were very small: but the facts already stated, clearly pointed to the sort of aid that it was necessary to attempt to procure for her.

"Leeches were applied to the tender part of the abdomen; and an ointment containing the hydriodate of potass was rubbed upon it. The action of the bowels was regulated by mild aperients; and, latterly, the hydriodate of potass was also administered internally. Two blisters were applied in the course of the treatment. The result of these remedies has been a restoration of the healthy feel of the abdomen; the swelling, tension, and hardness, having been altogether removed. The functions of the alimentary canal have become more natural; the pulse has diminished in frequency; the countenance has lost its expression of distress, and she has decidedly acquired flesh and strength." 231.

Our author expresses his conviction, that the above was not a case of chronic peritonitis, but "that it belonged unquestionably to that family of diseases, to which tubercles give the character." We confess that this part of the statement is not quite so clear to us, as we have seen many cases sufficiently similar to the above, which were cured on the principle of their being chronic peritoneal inflammation. By this, however, we do not wish to lessen the weight of Dr. Baron's evidence in favour of iodine, which, we have no doubt, will soon receive an extensive trial in tubercular diseases.

We must now bring this article to a close. Dr. Baron will not probably be satisfied that we have not gone into those controversial chapters, to which he will naturally be inclined to attach much importance. But it was quite impossible for us to give any thing like a connected view of the controversy, without occupying a space greater than the whole of this article now occupies—and even then we should have given little satisfaction to our readers, or to the parties concerned. Dr. Baron will perceive that we have assented to the main points of his doctrine, and if we do not attach so much importance to the views which he takes of tuberculous affections as he himself may think they demand, we are ready to express our admiration of the zeal, industry, and even talent, with which he prosecutes his pathological investigations.

The plates are admirably executed, and very delicately coloured. They convey a clear idea of the morbid structures they are meant to represent.

